# Perceived consequences of disseminating evidence on untreated recovery and moderate drinking after resolving alcohol concerns

**DOI:** 10.1186/s12954-026-01442-w

**Published:** 2026-03-30

**Authors:** John A. Cunningham

**Affiliations:** 1 National Addiction Centre, Institute of Psychiatry, Psychology and Neuroscience, Kings College London, London, UK; 2 Centre for Addiction and Mental Health, Toronto, Canada; 3 Department of Psychiatry, University of Toronto, Toronto, Canada

**Keywords:** Alcohol, Recovery, General public, Beliefs

## Abstract

**Aims:**

There is substantial evidence that many people recover from serious alcohol concerns without treatment and further, return to moderate alcohol consumption. The current study asks what people believe would happen if this evidence was widely disseminated.

**Design and methods:**

Participants (*N* = 3994), sampled to match the demographic characteristics of people in the UK, were recruited through the Prolific website. Participants were asked a series of questions about their current and past alcohol use. All participants were also asked their beliefs as to whether it would be problematic if evidence was disseminated regarding untreated and moderate drinking recoveries from serious alcohol problems.

**Results:**

Roughly half of the sample believed that it would make it harder for people to deal with their alcohol concerns if it was widely published that some people with serious alcohol problems can deal with their alcohol concerns without treatment and drink in a moderate/social manner after they have dealt with their concerns. Participants with prior to past year alcohol dependence were less likely to endorse that promulgating this information would be harmful compared to those who were never dependent or had lifetime alcohol dependence with past year symptoms.

**Discussion and conclusion:**

The results of this study contribute to the question of whether it is possible to promulgate the facts that untreated and moderate drinking recoveries are common without causing unanticipated consequences.

## Introduction

Formal treatments such as inpatient care and approaches such as Alcoholics Anonymous (AA) are widely accepted methods for addressing Alcohol Use Disorder (AUD). However, considerable evidence supports alternative pathways, notably untreated or “natural” recovery, where individuals resolve alcohol problems without intervention. Population surveys in multiple countries indicate that a substantial proportion of individuals who remit from alcohol dependence have not accessed specialty addiction services or AA [1–7]. Although natural recovery has been demonstrated as common and effective, public scepticism remains widespread. Cunningham et al. (1993) found that fewer than half of survey respondents believed individuals could overcome alcohol issues independently, contrasting sharply with public acceptance of untreated recovery from tobacco [8]. Professionals share this scepticism, often reinforced by clinical exposure to severe cases that typically require structured intervention [9].

Similarly, public acceptance of moderate drinking remains low, driven by enduring beliefs that abstinence is the only viable recovery outcome [10]. It is important to note that untreated recovery and moderation outcomes are conceptually distinct. Untreated recovery refers to resolution without formal specialty services, whereas moderation refers to a non-abstinent drinking pattern following resolution. Although these pathways may overlap, neither implies the other.

Broader paradigmatic perspectives, including abstinence-oriented frameworks, may also shape both public and professional responses to evidence regarding moderation and untreated recovery. In addition, conceptual models such as recovery capital and relational recovery emphasize the social context of recovery, suggesting that community beliefs may influence recovery trajectories. This is important because social stigma, including both public and self-stigma, significantly impacts recovery decisions. Stigma surrounding alcohol use diminishes individuals’ willingness to seek formal help and contributes to reluctance to publicly acknowledge untreated recovery or moderate drinking as acceptable outcomes [11].

Despite the empirical validity of untreated recovery and moderate drinking, limited research examines the public impact of disseminating evidence supporting these approaches. Prior research has focused primarily on whether individuals believe such recovery pathways are possible. However, belief in possibility is distinct from belief about the consequences of communicating that possibility. Public health decision-making often involves consideration of perceived unintended effects of messaging. Accordingly, this study examined whether participants believed disseminating evidence regarding untreated recovery and moderation would make it more difficult for others to address alcohol concerns. Alcohol was selected as the focus given its high prevalence in the UK and the established epidemiological literature on untreated recovery. It was predicted that beliefs regarding views of whether publishing such information would be harmful would mirror public beliefs that such recovery pathways were possible – that many participants would endorse that making this information widely available would make it harder for people with alcohol concerns to recover. In addition, the relationship of these beliefs to demographic and drinking characteristics were examined.

## Method

### Participants and procedures

This study was part of a broader investigation into alcohol dependence remission patterns in the UK.^7^ Participants were recruited via Prolific, an online research panel, and were sampled to match proportions of age, sex, and participant ethnicity found in the UK general population. The study invitation described it as a “short survey about drinking alcohol,” that it would take approximately 20–25 min to complete, and that they would be paid £4 into their Prolific account. Because recruitment materials referenced alcohol, individuals with greater personal interest or experience may have been more likely to participate. Eligible participants were aged 18 or older, with no restrictions based on drinking history.

### Questionnaire design

Alcohol use over the past year was measured using the consumption subscale of the Alcohol Use Disorders Identification Test (AUDIT-C) [12] Lifetime alcohol dependence was assessed using a scale adapted from the Ontario Drug Monitor, [13] aligned with ICD-10 criteria. The survey also asked questions regarding accessing treatment services [7]. However, because the prevalence of any treatment use ever was so low, this variable was not included in the analyses presented here (also, the inclusion of an ever treatment variable did not change the pattern of observed results). Participants responded to two questions about the public impact of sharing information about alternative pathways to recovery. They were asked whether it would be harder for people to seek help if it became widely known that some individuals recovered without treatment or resumed moderate drinking. Response options were “Yes,” “No,” or “Don’t know.” The questions did not operationally define “serious alcohol problems,” “treatment,” or “recovery,” and interpretations may therefore have varied across participants. The survey also included a series of questions on demographic characteristics – age, sex, education, marital status, and employment status. Finally, to ensure data quality, two attention checks were embedded in the survey and an item at the end of the survey asked participants to rate the truthfulness of their responses on a 7-point scale. Participants were told that their response to this question did not affect payment [14, 15].

### Statistical analysis

Participants who did not answer the attention check questions correctly, or who did not strongly endorse that they answered all questions honestly, were removed from the data set for analyses. Hierarchical logistic regressions were used to examine the predictors of participants’ attitudes toward the publication of recovery-related evidence that moderate drinking and untreated recoveries were possible. In Step 1, participant demographic characteristics were entered. In Step 2, current levels of alcohol consumption (AUDIT-C) and alcohol dependence status were entered (reference group—did not meet criteria to alcohol dependence; lifetime alcohol dependence with current symptoms; lifetime alcohol dependence with no past year symptoms). Because analyses were cross-sectional and descriptive; no causal inferences regarding communication effects can be drawn. Analyses were conducted using SPSS 29 software [16].

## Post-stratification weighting

An initial inspection of the sample’s age distribution revealed an overrepresentation of younger participants. To address this imbalance, post-stratification weighting was applied based on population estimates obtained from the Office for National Statistics for the United Kingdom as of July 2021 [17, 18]. Participants who identified outside of the binary categories of male or female were assigned a neutral weighting value of 1 to ensure their inclusion and accurate representation in subsequent analyses. Percentages, means, and standard deviations presented in the results are derived from the weighted data, while reported sample sizes reflect unweighted counts.

## Ethics approval

 Ethical approval for this study was obtained from the Research Ethics Board of King’s College London. Given the anonymous and web-based nature of this panel survey, informed consent was provided by participants electronically. Specifically, participants indicated consent by checking a box affirming their agreement to participate, subsequent to reviewing an information sheet outlining study details and participant rights.

## Results

A total of 3994 participants completed the survey and provided their Prolific ID. Of these, 200 were excluded because they did not strongly agree that they had answered all the questions truthfully and an additional 45 participants were excluded because they did not answer the attention check question correctly (*n* = 3749). Table 1 displays the demographic and drinking characteristics of these participants. For the two questions asking about beliefs, 53% believed it would make recovery harder if it were widely reported that individuals could return to moderate drinking after resolving alcohol problems. Further, 49% of participants believed that publishing information that some individuals recover without treatment would make recovery more difficult for others.

**Table 1:** Demographic characteristics of the full sample

Variable	*N* = 3749
Mean (SD) Age	48.2 (17.5)
% Male	47.6
% 19 or more years education	59.8
% Married/Common law/Same sex	60.2
% Full/Part-time employed	63.2
Mean (SD) AUDIT-C score	3.5 (2.6)
*Alcohol dependence status (ICD 10)*	
% Never dependent	80.3
% Lifetime with past year symptoms	13.4
% Lifetime with no past year symptoms	6.3
Would make it harder deal with alcohol concerns if widely published that ….	
% Moderate drinking possible	53.3
% Address alcohol concerns without treatment	48.9

Two hierarchical logistic regressions were performed to predict the beliefs that it would be harder for people to seek help if it became widely known that some individuals recovered without treatment or resumed moderate drinking. Demographic variables were entered in the first block and drinking severity measures were added in the second block. Table 2 shows the results of the two logistic regression analyses.

**Table 2:** Logistic regression analyses predicting whether it would make it harder deal with alcohol concerns if widely published that

Predictor	Impact if said moderate drinking possible			Impact if said treatment not required		
	χ^2^	β	EXP(β) (95% C.I)	χ^2^	β	EXP(β) (95% C.I.)
STEP 1	15.3**			35.8***		
Male		− 0.150*	0.861 (0.757–0.0980)		− 0.290***	0.748 (0.657–0.851)
Age		0.002	1.002 (0.997–1.006)		− 0.008***	0.992 (0.988–0.997)
19 + years education		− 0.026	0.975 (0.852–1.115)		− 0.135*	0.873 (0.763–1.000)
Married/Common Law		0.149*	1.160 (1.011–1.331)		0.155*	1.168 (1.018–1.340)
Employed (full or part time)		− 0.108	0.898 (0.778–1.036)		− 0.126	0.882 (0764-1.018)
STEP 2	25.3***			15.1**		
AUDIT-C score ^a^		− 0.050***	0.951 (0.923–0.979)		− 0.031*	0.969 (0.941–0.998)
Referent versus … ^b^						
Lifetime with past year symptoms		0.213	1.237 (0.997–1.535)		0.118	1.207 (0.937–1.498)
Lifetime with no past year symptoms		− 0.539***	0.584 (0.445–0.765)		− 0.449***	0.638 (0.485–0.841)

* *p* < 0.05. ** *p* < 0.01.** *p* < 0.001.***

^a^Alcohol use disorders test consumption index (score 0–12)

^b^Reference group consists of participants whose alcohol dependence status (ICD10) indicated that they had never been dependent on alcohol

The logistic regressions yielded some interpretable relationships linking demographics and drinking severity with beliefs that it would be harder for people to seek help if it became widely known that some individuals resumed moderate drinking or recovered without treatment. For the belief regarding the possibility of resuming moderate drinking, males and those who were not married or common law were less likely than other participants to believe that this information would be harmful. For the belief regarding the possibility of untreated recoveries, males, those who were younger, had more than 19 years of education, and who were not currently married or common law were less likely than other participants to believe that this information would be harmful. In addition, for both the beliefs that promulgating information about the possibility of moderate drinking and untreated recoveries, participants with higher AUDIT-C scores and those who met criteria for alcohol dependence in their lifetime but did not report any symptoms in the past year were less likely to believe that this information would be harmful.

## Discussion

This study examined public attitudes toward two topics often misunderstood in the context of alcohol addiction recovery: untreated recovery (natural recovery) and moderate drinking after resolving alcohol issues. Specifically, it explored whether people believe that sharing scientific evidence supporting these phenomena could hinder others from addressing their own alcohol problems. The study found that about half of participants were likely to view the publication of evidence supporting moderate drinking as potentially harmful, consistent with prior research showing scepticism toward non-abstinence-based recovery models [10]. Similarly, roughly half believed publishing evidence on untreated recovery could hinder recovery. These findings reflect perceived consequences rather than demonstrated effects of dissemination.

Gender differences were observed. Participants identifying as male were less likely to believe than other participants that publishing evidence on both moderate drinking and untreated recovery would negatively impact others. Further, participants who were married or in a common law relationship were more likely to believe that releasing information about the possibility of moderated drinking and untreated recoveries would be harmful. Finally, those who were younger and had more than 19 years of education were less likely to believe that information regarding the possibility of untreated recovery would be harmful.

Participants with a history of alcohol dependence—but no recent symptoms—were more likely to support the publication of evidence on both moderate drinking and untreated recovery. One possible interpretation is that personal experience with resolution of alcohol problems may influence perceptions of communication risk; however, this inference cannot be confirmed within the present design. Similarly, those with higher levels of current alcohol consumption were less likely to believe that publishing these findings would be harmful. As with other cross-sectional associations observed here, causal direction and underlying mechanisms cannot be inferred. The mechanisms underlying these associations warrant further investigation.

### Harm reduction implications

From a harm reduction perspective, dissemination of evidence acknowledging multiple recovery pathways may be viewed as consistent with approaches that recognize heterogeneity in trajectories. However, the present findings indicate that a substantial minority of respondents perceive such dissemination as potentially harmful. Communication strategies that reference untreated recovery or moderation may therefore require careful framing to avoid misinterpretation, particularly when addressing severe alcohol-related problems.

### Limitations

The study has several limitations. First, online panel recruitment limits generalizability despite quota sampling and post-stratification weighting. The sample cannot be considered fully representative of the UK population. Second, as a retrospective survey, it may be affected by recall bias—particularly in participants’ reports of past alcohol use and consequences. Third, the cross-sectional design precludes inference regarding causal relationships between participant characteristics and perceived harmfulness. Fourth, the recruitment message mentioned alcohol, which may have attracted participants with a greater personal interest in the topic, possibly skewing responses. Fourth, single-item measures were used to assess attitudes, which may lack nuance. Additionally, the response options (“yes,” “no,” “don’t know”) limited the range of expression, potentially overstating the number of people perceived as indifferent or supportive. Lastly, attitudes were measured without contextual framing. Participants were not provided with standardized definitions of “serious alcohol problems,” “treatment,” or “recovery,” and interpretations may therefore have varied. Future studies could provide background information—such as news stories or official reports—before collecting responses to better simulate real-world exposure and more accurately gauge public reactions. In addition, future research may benefit from multi-item measures, experimental manipulation of severity framing, and qualitative approaches to clarify how respondents interpret dissemination-related questions.

## Conclusion

Most existing studies focus on the effectiveness and prevalence of untreated recovery and moderate drinking. This research adds a new dimension by exploring whether the public perceives that promoting such evidence could discourage others from seeking help or maintaining recovery. In doing so, it fills a gap in the literature and offers a foundation for further exploration of how recovery narratives are received. Further, the study analysed public opinion not just generally, but also by alcohol use level and dependence history. Findings suggest that attitudes are influenced by personal experience and consumption patterns. In the UK, limited research exists on public acceptance of non-traditional recovery models. This study helps contextualize these issues within the UK’s cultural landscape and offers insight for public health messaging and policy.

## Data Availability

Available from the corresponding author on reasonable request.

## References

[1] Dawson DA. Correlates of past–year status among treated and untreated persons with former alcohol dependence: United States, 1992. Alcoholism: Clin Experimental Res. 1996;20(4):771–9. 10.1111/j.1530-0277.1996.tb01685.x 8800398

[2] Dawson DA, Grant BF, Stinson FS, Chou PS, Huang B, Ruan WJ. Recovery from DSM-IV alcohol dependence: United States, 2001–2002. Addiction Mar. 2005;100(3):281–92. 10.1111/j.1360-0443.2004.00964.x 15733237

[3] Rumpf HJ, Bischof G, Hapke U, Meyer C. Studies on natural recovery from alcohol dependence: Sample selection bias by media solicitation? Addiction. 2000;95:765–75. 10885051 10.1046/j.1360-0443.2000.95576512.x

[4] Sobell LC, Cunningham JA, Sobell MB. Recovery from alcohol problems with and without treatment: prevalence in two population surveys. Am J Public Health Jul. 1996;86(7):966–72. 10.2105/ajph.86.7.966. 10.2105/ajph.86.7.966 PMC1380437 8669520

[5] Blomqvist J, Cunningham JA, Wallander L, Collin L. Att Förbättra Sina Dryckesvanor – om olika monster för förändring och om vad vården betyder (Improving one’s drinking habits – on different patterns of change and on the role of alcohol treatment). 2007.

[6] Cunningham JA, Blomqvist J, Koski-Jännes A, Cordingley J, Callaghan R. Characteristics of former heavy drinkers: results from a natural history of drinking general population survey. Contemp Drug Probl. 2004;31(Summer):357–69.

[7] Cunningham JA, Schell C, Walker H, Godinho A. Patterns of remission from alcohol dependence in the United Kingdom: results from an online panel general population survey. Substance Abuse Treat Prev Policy Jan. 2024;4(1):3. 10.1186/s13011-023-00588-1. 10.1186/s13011-023-00588-1 PMC10768276 38178169

[8] Cunningham JA, Sobell LC, Chow VM. What’s in a label? The effects of substance types and labels on treatment considerations and stigma. J Stud Alcohol. 1993;54:693–9. 8271805 10.15288/jsa.1993.54.693

[9] Samuelsson E, Blomqvist J, Christophs I. Addiction and recovery: perceptions among professionals in the Swedish Treatment System. Nord Stud Alcohol Dr. 2013;20:51–66.

[10] Cunningham JA, Blomqvist J, Cordingley J. Beliefs about drinking problems: results from a general population telephone survey. Addict Behav Jan. 2007;32(1):166–9. 10.1016/j.addbeh.2006.03.011 16626882

[11] Rundle SM, Cunningham JA, Hendershot CS. Implications of addiction diagnosis and addiction beliefs for public stigma: a cross-national experimental study. Drug Alcohol Review Jan. 2021;25. 10.1111/dar.13244. 10.1111/dar.13244 33493359

[12] Babor TF, De La Fuente MF, Saunders JB, Grant M. AUDIT - The alcohol use disorders identification test: guidelines for use in primary health care. World Health Organization; 1989.

[13] Adlaf EM, Ivis F, Bondy S, Rehm J, Room R, Walsh G. The Ontario Drug Monitor, 1996: Technical Report. 1997. 132.

[14] Godinho A, Kushnir V, Cunningham JA. Unfaithful findings: identifying careless responding in addictions research. Addiction Jun. 2016;111(6):955–6. 10.1111/add.13221. 10.1111/add.13221 26662631

[15] Godinho A, Schell C, Cunningham JA. Out damn bot, out: Recruiting real people into substance use studies on the internet. Subst Abus Dec. 2020;10:41:2–5. 10.1080/08897077.2019.1691131. 10.1080/08897077.2019.1691131 31821108

[16] IBM SPSS Statistics for Windows, Version 29.0. IBM; 2022.

[17] Office for National Statistics. Data from: estimates of the population for the UK, England, Wales, Scotland and Northern Ireland 2022.

[18] Royal KD. Survey research methods: a guide for creating post-stratification weights to correct for sample bias. Educ Health Prof. 2019;2:48–50.

